# Supplementary material for: Rhodiola rosea Reduces Intercellular Signaling in Campylobacter jejuni
Source: Antibiotics (Basel). 2022 Sep 8;11(9):1220. doi: 10.3390/antibiotics11091220 (PMC9494958; doi:10.3390/antibiotics11091220)
Supplement: Supplementary file 1 [file antibiotics-11-01220-s001.zip › antibiotics-1861612-supplementary.pdf]

Table S1. Identified compounds in the ethanolic extract from *R. rosea* "Mattmark".

No.	tR (min)	$[M-H]^-$ (m/z)	MS^n [parent ion], m/z	UV λ_{max}	Identified compounds	Compound group
1	1.60	169	MS^2 [169]: 125	215, 272	Gallic acid	Benzoic acid
2	3.06	331	/	/**	Galloyl glucose	Phenolic glycoside
3	3.11	258	MS^2 [304]*: 258, 179,161<br>MS^3 [179]: 161, 143, 131, 119, 89	209	Rhodiocyanoside A	Cyanogenic glycoside
4	4.17	260	MS^2 [306]*: 260, 188, 161<br>MS^3 [260]: 188, 161	/**	Lotaustralin	Cyanogenic glycoside
5	4.61	297	MS^2 [343]*: 297, 135	266	Phenolic hexoside	Phenolic hexoside
6	4.90	299	MS^2 [345]*: 299<br>MS^3 [299]: 179, 161, 143, 131, 119, 113, 101	221, 276	Salidroside	Phenylethyl glycoside
7	5.51	761	MS^2 [761]: 635, 609, 593, 591, 473, 483, 423<br>MS^3 [423]: 379, 297, 283, 243	207, 273	Epigallocatechin-epigallocatechin gallate (EGC-EGCG)	Proanthocyanidin dimer
8	7.27	913	MS^2 [913]: 761, 743, 591, 573, 423<br>MS^3 [761]: 379	205, 275	Epigallocatechin gallate-epigallocatechin gallate (EGCG-EGCG)	Proanthocyanidin dimer
9	7.73	457	MS^2 [457]: 331, 305, 169<br>MS^3 [169]: 125	207, 274	Epigallocatechin-3-O-gallate	Flavanol (Catechin)
10	8.92	415	MS^2 [461]*: 415<br>MS^3 [415]: 191, 149, 131	/**	2-Phenylethyl-vicianoside	Phenylethyl glycoside
11	10.52	427	MS^2 [473]*: 427, 293<br>MS^3 [427]: 293, 149	251	Rosarin	Cinnamyl alcohol glycoside
12	10.77	465	MS^2 [511]*: 465<br>MS^3 [465]: 333, 293, 191, 149	/**	Rhodioloside E	Acyclic alcohol glycoside
13	10.88	427	MS^2 [473]*: 427, 293<br>MS^3 [427]: 293, 149	251	Rosavin	Cinnamyl alcohol glycoside
14	11.18	427	MS^2 [473]*: 427, 293<br>MS^3 [427]: 293, 149	252	Cinnamyl-pentosyl-hexoside	Cinnamyl alcohol glycoside
15	11.47	331	MS^2 [377]*: 331, 179<br>MS^3 [179]: 161, 143, 131, 119, 113, 89	193, 262	Rosiridin	Acyclic alcohol glycoside
16	14.76	609	MS^2 [609]: 463, 301<br>MS^3 [301]: 301, 283, 255, 229, 211, 201	276, 332, 384	Rhodiosin (herbacetin-7-O-glucorhamnoside)	Flavonol glycoside
17	15.10	447	MS^2 [447]: 301<br>MS^3 [301]: 301, 283, 255, 229, 211, 201	276, 329, 384	Rhodionin (herbacetin-7-O-α-L-rhamnopyranoside)	Flavonol glycoside
18	18.66	479	/	280, 330, 338	Rhodiolin	Flavolignan

* Fragmentation of $[M-H+HCOOH]^-$ as parent ion in MS^2 is indicated by an asterisk, in all other cases fragment ions of $[M-H]^-$ as parent ion are presented;
** Not detected in the UV chromatogram due to the low concentration; / no further fragment ions observed (low concentration)

Table S2. Identified compounds in the ethanolic extract from *R. rosea* "Rosavine".

No.	tR (min)	[M-H] (m/z)	MS^n (m/z)	UV λmax	Identified compounds	Compound group
1	1.62	169	MS^2 [169]: 125	215, 269	Gallic acid	Benzoic acid
2	3.07	331	/	209	Galloyl glucose	Phenolic glycoside
3	3.10	258	MS^2 [304]*: 258, 179,161<br>MS^3 [179]: 161, 143, 131, 119, 89	209	Rhodiocyanoside A	Cyanogenic glycoside
4	4.15	260	MS^2 [306]*: 260, 188, 161<br>MS^3 [260]: 188, 161	/**	Lotaustralin	Cyanogenic glycoside
5	4.88	299	MS^2 [345]*: 299<br>MS^3 [299]: 179, 161, 143, 131, 119, 113, 101, 89	221, 276	Salidroside	Phenylethyl glycoside
6	5.51	761	MS^2 [761]: 635, 609, 593, 575, 483, 423<br>MS^3 [423]: 379, 297, 283, 243	207, 276	Epigallocatechin-epigallocatechin gallate (EGC-EGCG)	Proanthocyanidin dimer
7	7.27	913	MS^2 [913]: 761, 743, 591, 573, 423<br>MS^3 [761]: 379	205, 276	Epigallocatechin gallate-epigallocatechin gallate (EGCG-EGCG)	Proanthocyanidin dimer
8	7.72	457	MS^2 [457]: 331, 305, 169<br>MS^3 [169]: 125	207, 275	Epigallocatechin-3-O-gallate	Flavanol (Catechin)
9	8.92	415	MS^2 [461]*: 415<br>MS^3 [415]: 191, 149, 131	/**	2-Phenylethyl-vicianoside	Phenylethyl glycoside
10	9.63	609	MS^2 [609]*: 463<br>MS^3 [463]: 301, 300	275, 326, ?	Rhodionidin (herbacetin-7-O-rhamnosyl-8-O-glucoside)	Flavonol glycoside
11	10.51	427	MS^2 [473]*: 427, 293<br>MS^3 [427]: 293, 149	251	Rosarian	Cinnamyl alcohol glycoside
12	10.80	465	MS^2 [511]*: 465<br>MS^3 [465]: 333, 293, 191, 149	/**	Rhodioloside E	Acyclic alcohol glycoside
13	10.88	427	MS^2 [473]*: 427, 293<br>MS^3 [427]: 239, 149	251	Rosavin	Cinnamyl alcohol glycoside
14	11.18	427	MS^2 [473]*: 427, 293<br>MS^3 [427]: 239, 149	251	Cinnamyl-pentosyl-hexoside	Cinnamyl alcohol glycoside
15	11.46	331	MS^2 [377]*: 331, 179<br>MS^3 [179]: 161, 143, 131, 119, 89	193, 262	Rosiridin	Acyclic alcohol glycoside
16	14.76	609	MS^2 [609]: 463, 301<br>MS^3 [301]: 301, 283, 255, 229, 211, 201	276, 332, 384	Rhodiosin (herbacetin-7-O-glucorhamnoside)	Flavonol glycoside
17	15.10	447	/	276, 330, 384	Rhodionin (herbacetin-7-O-α-L-rhamnopyranoside)	Flavonol glycoside

* Fragmentation of $[M-H+HCOOH]^-$ as parent ion in MS^2 is indicated by an asterisk, in all other cases fragment ions of $[M-H]^-$ as parent ion are presented;
** Not detected in the UV chromatogram due to the low concentration; / no further fragment ions observed (low concentration)

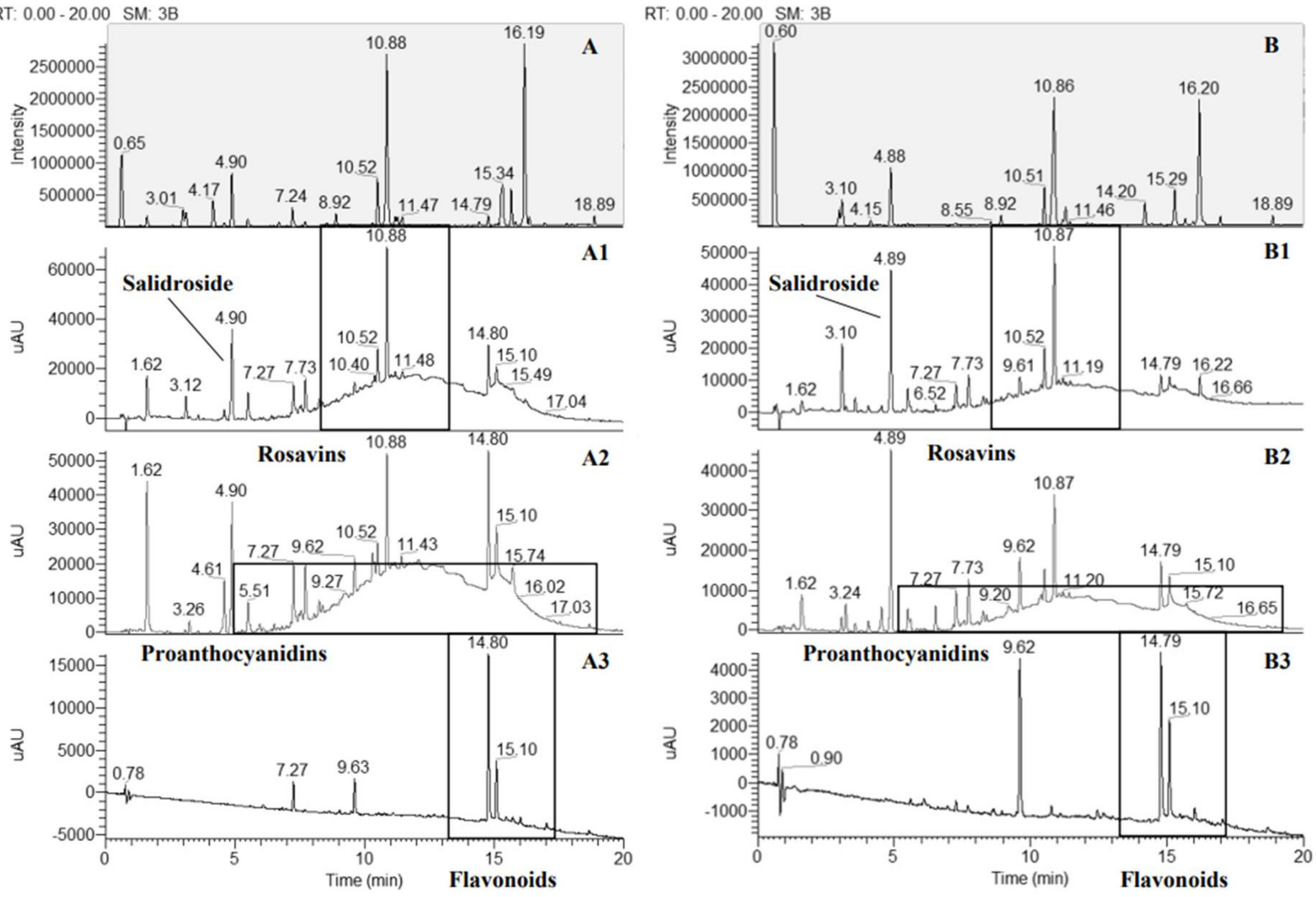

Figure S1. The base peak (**A**: “Mattmark”; **B**: “Rosavine”) and UV chromatograms (**A1**; **B1**: total scan; **A2**; **B2**: 276 nm; **A3**; **B3**: 360 nm) from UHPLC-PDA-ESI-MS analysis of *R. rosea* ethanolic extracts.

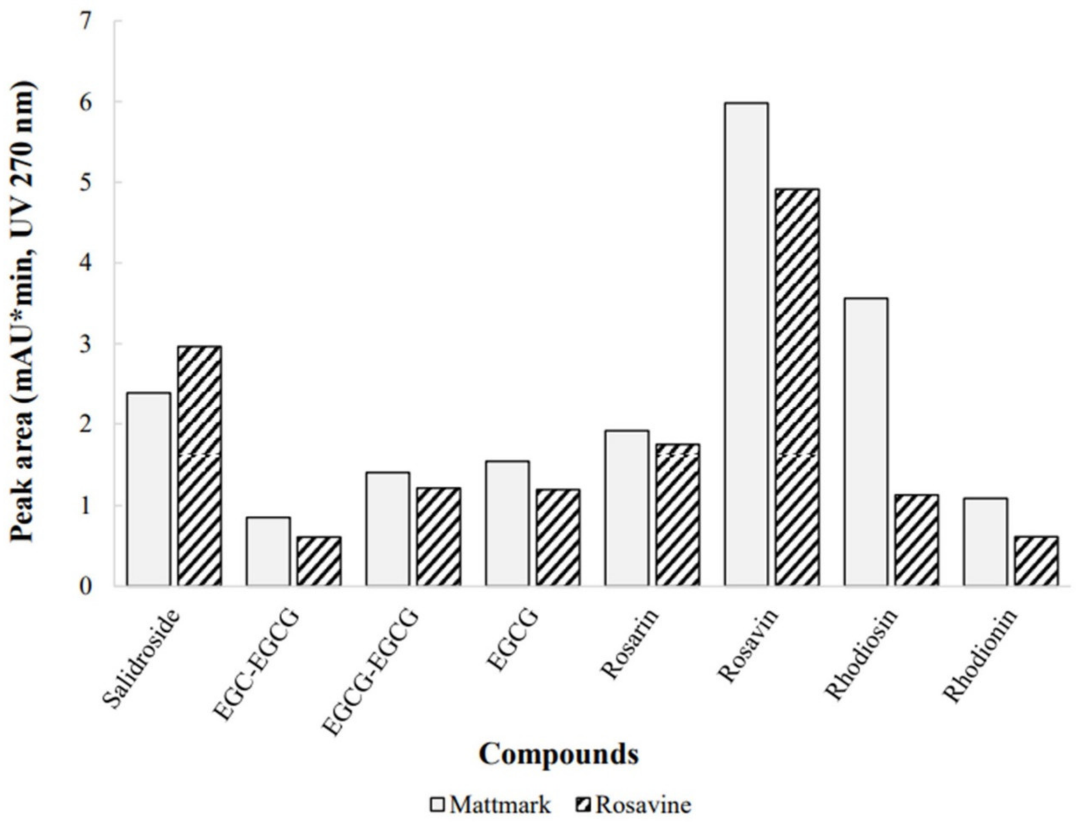

Figure S2. Comparison of peak areas at UV 270 nm of the crude ethanolic extracts of *R. rosea* roots “Mattmark” and “Rosavine”. Samples were analyzed at a concentration of 5 mg/mL.

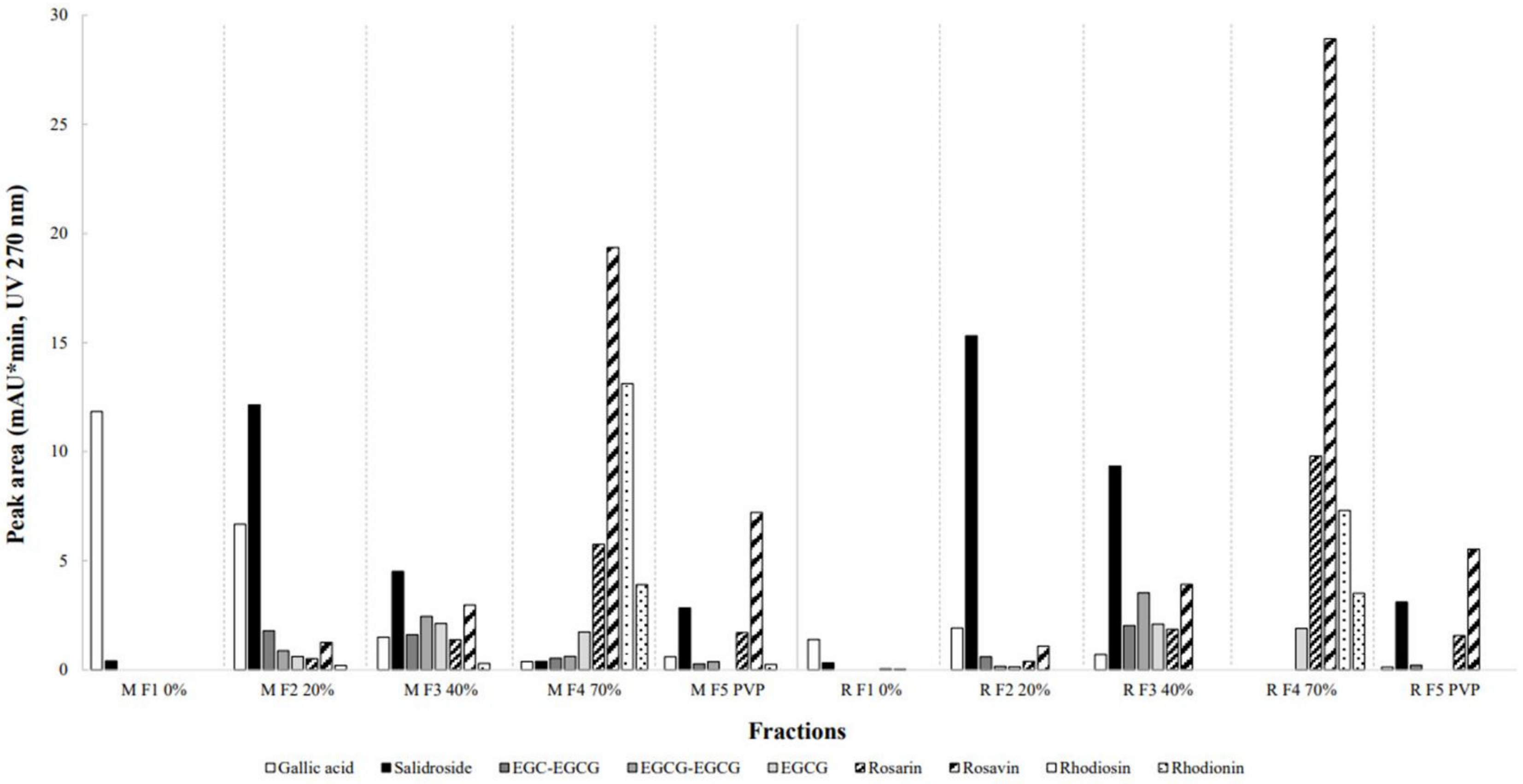

Figure S3. Comparison of peak areas at UV 270 nm of fractions F1 to F5 of “Mattmark” (M) and “Rosavine” (R). Samples were analyzed at a concentration of 5 mg/mL.
